# *Trichinella spiralis* HSP70 Mediates Mice Immune Responses via TLR2/MyD88/ERK Signaling Pathway

**DOI:** 10.1155/tbed/5533482

**Published:** 2026-05-27

**Authors:** Jiajun Feng, Yuan Li, Muhammad Azhar Memon, Yuheng Zhang, Mingmin Lu, Xiaokai Song, Lixin Xu, Ruofeng Yan

**Affiliations:** ^1^ MOE Joint International Research Laboratory of Animal Health and Food Safety, College of Veterinary Medicine, Nanjing Agricultural University, Nanjing, 210095, Jiangsu, China, njau.edu.cn

**Keywords:** HSP70, immune protection, MAPK, NF-κB, TLR2, *Trichinella spiralis*

## Abstract

Trichinellosis is a globally important zoonotic parasitic disease that poses a significant threat to public health and causes substantial economic losses. *Trichinella spiralis* heat shock protein 70 (Ts‐HSP70) has been identified as a potential vaccine candidate with immunomodulatory properties. However, the underlying mechanisms by which Ts‐HSP70 regulates host immune responses remain incompletely understood. In this study, we investigated the role of Ts‐HSP70 in modulating macrophage functions and explored its involvement in Toll‐like receptor 2 (TLR2)‐mediated signaling. The interaction between Ts‐HSP70 and mouse TLR2 was examined using co‐immunoprecipitation (Co‐IP) and fluorescence colocalization assays. Recombinant Ts‐HSP70 (rTs‐HSP70) was used to stimulate RAW264.7 macrophages in vitro, and its effects on cell proliferation, phagocytosis, nitric oxide (NO) production, reactive oxygen species (ROS) generation, and cytokine mRNA expression were evaluated. rTs‐HSP70 significantly enhanced macrophage proliferation and phagocytic activity, increased NO and ROS production, and upregulated the mRNA expression of IL‐1β, IL‐6, TNF‐α, and IL‐10. In addition, rTs‐HSP70‐induced activation of NF‐κB and MAPK signaling pathways. Functional inhibition assays further suggested that ERK activation was partially dependent on the TLR2/MyD88 pathway. Furthermore, rTs‐HSP70 encapsulated in poly(lactic‐co‐glycolic acid) (PLGA) nanoparticles elicited specific antibody responses and conferred partial protection in mice, as evidenced by a 28.0% reduction in muscle larval burden. Collectively, these findings indicate that Ts‐HSP70 modulates macrophage immune functions and may contribute to host defense through TLR2‐associated signaling, providing insights into its potential application in vaccine development against trichinellosis.

## 1. Introduction

Trichinellosis is a severe zoonotic parasitic disease primarily transmitted to humans through the consumption of meat products containing infective *Trichinella* larvae. It imposes a significant burden on both public health and the global economy. Epidemiological estimates indicate that ~10 million people were infected in the 1990s [1–3]. The economic impact is substantial, with annual *Trichinella* monitoring costs estimated at approximately $1 billion in the United States and at $570 million in the European Union [3, 4]. Vaccination is a crucial strategy for preventing infectious diseases, offering benefits including long‐term immune protection, reduced emergence of drug‐resistant strains, and fewer drug residues in meat products. Despite this, no commercial vaccine is currently available against trichinellosis, underscoring the urgent need to screen novel candidate antigens.

Innate immunity constitutes the first line of defense against pathogens, relying on pattern recognition receptors (PRRs) to identify pathogens. Among these, Toll‐like receptors (TLRs) represent a major class of PRRs [5, 6]. Upon recognition of pathogen‐associated molecular patterns (PAMPs), most TLRs undergo dimerization, leading to conformational changes in their TIR domains that facilitate the recruitment of adaptor proteins (such as TIRAP, MyD88, and TRIF). These adaptor proteins then activate downstream signaling cascades, including NF‐κB and MAPK pathways, ultimately inducing the expression and release of type I interferons, inflammatory factors, chemokines, and costimulatory molecules to initiate innate immunity and bridge it to adaptive immunity [7–9]. During *T. spiralis* infection, alterations in the expression of various TLRs have been observed in the host [10–12]. Notably, TLR2 exhibits broad ligand specificity and can form heterodimers with multiple other TLRs [13–15], suggesting its potential role in mediating the immune response against *T. spiralis* infection.

The heat shock protein (HSP) family has been extensively studied for decades in various fields, including cellular stress response, immune adjuvant development, cancer, and autoimmune diseases [16–18]. HSP70 is a highly conserved member of the HSP family. In addition to being upregulated during cellular stress and acting as a molecular chaperone to ensure proper protein folding, it can also function as an extracellular immune signaling molecule [19, 20]. Wang et al. [21] cloned and expressed the Ts‐HSP70 protein and conducted a protective efficacy trial in mice using Freund’s adjuvant. They found that rTs‐HSP70 provided partial immuno‐protection, reducing the muscle larval burden by 37%. Subsequent experiments confirmed that HSP70 induces dendritic cell (DC) maturation via TLR2 and TLR4, thereby eliciting immune protection against *T. spiralis* in mice [22, 23].

Despite these findings, the immunomodulatory mechanism of Ts‐HSP70 remains incompletely understood. It is unclear whether Ts‐HSP70 activates specific downstream signaling pathways following TLR engagement and how it influences immune cells beyond DCs. Given the central role of macrophages in orchestrating innate and adaptive immune responses, elucidating the effects of Ts‐HSP70 on macrophage function is of particular importance. Therefore, the present study aimed to investigate the interaction between Ts‐HSP70 and TLR2 and to evaluate its role in modulating macrophage immune responses, with a focus on NF‐κB and MAPK signaling pathways.

## 2. Materials and Methods

### 2.1. Animals, Parasites, and Cells

ICR mice were housed under specific pathogen‐free (SPF) conditions at the Laboratory Animal Center of Nanjing Agricultural University. The *T. spiralis* pig‐derived isolate (International Code: ISS534) was originally obtained from Henan, China, and is maintained in our laboratory. All animal experiments and procedures were approved by the Laboratory Animal Welfare and Ethics Committee of Nanjing Agricultural University (Approval Number: NJAU.No20240718141).

### 2.2. Construction of Eukaryotic Expression Plasmids

Total RNA was isolated from RAW264.7 cells and *T. spiralis* muscle larvae using the RNA isolater Total RNA Extraction Reagent (Vazyme Biotech, Nanjing, China). Complementary DNA (cDNA) was synthesized by the reverse transcription polymerase chain reaction (RT‒PCR). Primers for TLR2 (GenBank NM_011905.3) ectodomain and Ts‐HSP70 (GenBank AY046874.2) were designed with HA‐tag and FLAG‐tag coding sequences, respectively. The primer sequences for TLR2‐pCAGGS and HSP70‐pCAGGS are listed in Table 1. The targeted genes were amplified by PCR (Phusion Plus Green PCR Master Mix, Thermo Fisher Scientific, Waltham, USA), and the products were gel‐purified using the Gel Extraction Kit (OMEGA, Knoxville, GA, USA), following the manufacturers’ protocols. The purified gene fragments TLR2 ectodomain and Ts‐HSP70 were digested with appropriate restriction enzymes and ligated into the pCAGGS vector, constructing the recombinant plasmids pCAGGS‐TLR2‐HA and pCAGGS‐HSP70‐FLAG.

**Table 1:** Primers used for plasmid construction.

Primer	Primer (5′‐3′)
TLR2‐pCAGGS	Forward: GGGGTACCGCCACCATGCAGGAGTCTCTGTCATGTGAT
	Reverse: CCGCTCGAGCTAAGCGTAGTCTGGGACGTCGTA TGGGTACTGGTGACATTCCAAGACGGA
HSP70‐pCAGGS	Forward: CGGAATTCGCCACCATGGGTATTGACCTTGGAACAACA
	Reverse: CCGCTCGAGTCACTTGTCATCGTCGTCCTTGT AATCCAGTTCATCTTTTTCTTC
pCold‐HSP70	Forward: CCGCTCGAGGGTATTGACCTTGGAACAACA
	Reverse: CGGAATTCTCACAGTTCATCTTTTTCTTCAGC

### 2.3. Co‐Immunoprecipitation (Co‐IP) Assay

Co‐IP was performed to verify the interaction between HSP70 and TLR2. HEK293T cells were resuscitated from cryopreservation and cultured in Dulbecco’s Modified Eagle Medium (DMEM) supplemented with 10% fetal bovine serum (FBS) at 37°C under 5% CO_2_ until stable growth and morphology were achieved. Subsequently, cells were seeded into 60 mm culture dishes. Cotransfection of pCAGGS‐TLR2‐HA and pCAGGS‐HSP70‐FLAG plasmids was performed using Lipofectamine 2000 (Invitrogen Biotechnology, Carlsbad, CA, USA) according to the manufacturer’s protocol. After 24 h of incubation, cells were collected and lysed with NP‐40 lysis buffer (Beyotime, Shanghai, China). For the experimental groups, lysates were incubated with FLAG‐Tag Rabbit mAb (ABclonal, Wuhan, China) or HA‐Tag Rabbit mAb (ABclonal, Wuhan, China), whereas the control group was incubated overnight with Normal Rabbit IgG (Abmart, Shanghai, China) at 4°C on a shaker. Subsequently, rProtein A/G Magarose Beads (Smart Lifesciences, Changzhou, China) were added, followed by 4 h of incubation. After three washes performed according to the manufacturer’s protocol, protein complexes were eluted for Western blot analysis. To eliminate interference from heavy‐ and light‐chain antibodies, species‐mismatched antibodies were used for Western blot analysis. FLAG‐Tag Mouse mAb and HA‐Tag Mouse mAb (Abmart, Shanghai, China) were used to detect protein complexes in each group.

### 2.4. Colocalization Analysis

HEK293T cells were seeded on glass coverslips and cotransfected, as described in Section 2.3. After 24 h of incubation, coverslips were collected, and cells were fixed, permeabilized, and blocked with 5% skim milk at 37°C for 1 h. Samples were incubated with primary antibodies, HA‐Tag Rabbit mAb and FLAG‐Tag Mouse mAb, at 4°C overnight. After primary antibody removal, secondary antibodies FITC‐conjugated goat anti‐mouse IgG (H + L) and Cy3‐conjugated goat anti‐rabbit IgG (H + L) (ABclonal, Wuhan, China) were applied at 37°C for 1 h. Finally, coverslips were mounted with antifade mounting medium containing Hoechst 33342 (Beyotime, Shanghai, China) and examined using a laser scanning confocal microscope.

### 2.5. Recombinant Ts‐HSP70 Protein Preparation

The Ts‐HSP70 gene was amplified as described in Section 2.2 using primers pCold‐HSP70 (sequences in Table 1). The Ts‐HSP70 gene was cloned into the pCold‐Ⅰ vector to construct the recombinant plasmid pCold‐HSP70, which was transformed into competent *Escherichia coli* (*E. coli*) BL21 (DE3) (Sangon Biotech Shanghai, China). The expression of rTs‐HSP70 was induced with isopropyl‐β‐D‐thiogalactopyranoside (IPTG) under low temperature conditions to enhance soluble protein yield. The expression of the recombinant protein was confirmed by SDS‐PAGE and Western blot analysis. The rTs‐HSP70 protein was purified using a His‐Trap FF affinity column (Cytiva, Marlborough, MA, USA). Endotoxins were subsequently removed with the ToxinEraser Endotoxin Removal Kit (GenScript, Nanjing, China), which employs a modified polymyxin B‐based method for specific endotoxin clearance. Residual endotoxin levels were then measured with the Kinetic Turbidimetric LAL Endotoxin Assay Kit (Beyotime, Shanghai, China).

A Western blot was conducted to validate recombinant protein expression using rat anti‐*T. spiralis* serum, serum from naive rats (negative control), and His‐tag monoclonal antibody (Proteintech, Wuhan, China) as primary antibodies. Briefly, purified rTs‐HSP70 was separated by SDS‐PAGE and transferred onto polyvinylidene difluoride (PVDF) membranes (Millipore, Billerica, MA, USA), followed by blocking with 5% skim milk at 37°C for 2 h. Membranes were subsequently incubated with the following primary antibodies: rat anti‐*T. spiralis* serum (1:500), serum from naive rats (1:500), or anti‐his‐tag mAb (1:10,000) at 37°C for 1 h. After washing, membranes were treated with species‐matched HRP‐conjugated secondary antibodies: goat anti‐rat IgG (H + L) or goat anti‐mouse IgG (H + L) at 37°C for 1 h. Protein signals were visualized using an enhanced chemiluminescence (ECL) detection system (Tiangen, Beijing, China).

### 2.6. Effect of rTs‐HSP70 on Cellular Proliferation in Mouse Macrophages

RAW264.7 cells were cultured, as described in Section 2.3. Cells were adjusted to a density of 1 × 10^5^ cells/mL and seeded into 96‐well plates. rTs‐HSP70 was added at concentrations of 5, 10, 20, and 40 μg/mL, with the PBS group as a control. After 12 h of stimulation, 10 μL CCK‐8 solution (Beyotime, Shanghai, China) was added to each well, followed by 2 h of incubation. Absorbance was measured at 450 nm using a microplate reader.

### 2.7. Effect of rTs‐HSP70 on Phagocytic Activity in Mouse Macrophages

RAW264.7 cells were adjusted to a density of 1 × 10^6^ cells/mL and seeded into 24‐well plates. Cells were treated with rTs‐HSP70 as grouped in Section 2.6, followed by a 12 h stimulation. FITC‐dextran (Sigma–Aldrich, Shanghai, China; 500 μg/mL final concentration) was added and incubated for an additional 1 h. Cells were collected, washed with PBS, and analyzed for mean fluorescence intensity using flow cytometry.

### 2.8. Effect of rTs‐HSP70 on ROS and NO Production in Mouse Macrophages

RAW264.7 cells were adjusted to 1 × 10^6^ cells/mL and seeded into culture plates. Cells were treated with rTs‐HSP70 as grouped in Section 2.6, followed by 18 h of stimulation. Intracellular ROS levels and NO production were measured using a ROS assay kit and a NO assay kit (Beyotime, Shanghai, China), respectively, according to the manufacturer’s protocols.

### 2.9. Effects of rTs‐HSP70 on Cytokine mRNA Expression in Mouse Macrophages

RAW264.7 cells were adjusted to a density of 1 × 10^6^ cells/mL and seeded into 12‐well plates. Cells were treated with rTs‐HSP70 as grouped in Section 2.6, followed by 6 h of stimulation. Total RNA was extracted and subjected to reverse transcription quantitative PCR (RT‐qPCR) to quantify the mRNA levels of inflammatory cytokines IL‐1β, IL‐6, TNF‐α, and IL‐10. Data were analyzed using the 2^−ΔΔCt^ method, with qPCR primers listed in Table 2 [24, 25].

**Table 2:** qPCR primers.

Primer	Sequence (5′‐3′)
IL‐6 F	AGACAAAGCCAGAGTCCTTCA
IL‐6 R	GCCACTCCTTCTGTGACTCC
IL‐10 F	GACAACATACTGCTAACCGACTCC
IL‐10 R	TCCTTGATTTCTGGGCCATGC
IL‐1β F	GCAACTGTTCCTGAACTCAACT
IL‐1β R	ATCTTTTGGGGTCCGTCAACT
TLR2 F	CGTTGTTCCCTGTGTTGCTG
TLR2 R	CAGAGCTGGCGTCTCCATAG
TNF‐α F	GATCGGTCCCCAAAGGGATG
TNF‐α R	GGTGGTTTGTGAGTGTGAGGG
MyD88 F	CGGAACTTTTCGATGCCTTTAT
MyD88 R	CACACACAACTTAAGCCGATAG
GAPDH F	CATCACTGCCACCCAGAAGACTG
GAPDH R	ATGCCAGTGAGCTTCCCGTTCAG

*Note:* GAPDH gene: glyceraldehyde‐3‐phosphate dehydrogenase gene; IL‐6: Interleukin 6 gene; IL‐1β: Interleukin 1β gene; IL‐10: Interleukin 10 gene; TNF‐α: Tumor Necrosis Factor‐alpha gene; TLR2: Toll‐like receptor 2 gene; MyD88: Myeloid differentiation primary response gene 88 gene.

### 2.10. Effect of rTs‐HSP70 on TLR2 and MyD88 Expression in Mouse Macrophages

RAW264.7 cells were adjusted to a density of 1 × 10^6^ cells/mL and seeded into 12‐well plates. Cells were treated with rTs‐HSP70 as grouped in Section 2.6, followed by a 12 h stimulation. Total proteins were extracted using RIPA lysis buffer (Invent, Beijing, China) and subjected to Western blot analysis.

### 2.11. Effects of rTs‐HSP70 on NF‐κB and MAPK Pathways in Mouse Macrophages

RAW264.7 cells were adjusted to a density of 1 × 10^6^ cells/mL and seeded into 12‐well plates. Cells were treated with 5 μg/mL rTs‐HSP70 and stimulated for 0, 15, and 30 min. Total proteins were extracted using RIPA lysis buffer and subjected to Western blot analysis.

### 2.12. TLR2/MyD88 Dependence Validation

To verify whether rTs‐HSP70 activates the NF‐κB and MAPK pathways via the TLR2/MyD88 signaling pathway, we pretreated cells with a TLR2‐specific antibody (Novus Biologicals, Centennial, CO, USA) and the MyD88 inhibitor T6167923 (MedChemExpress, Monmouth Junction, NJ, USA). Briefly, RAW264.7 cells were adjusted to 1 × 10^6^ cells/mL and treated with the TLR2‐specific antibody (20 μg/mL). After incubation at 37°C for 1 h, the cells were stimulated with 1 μg/mL rTs‐HSP70 for 15 min and then collected for Western blot analysis. The control group received an equivalent amount of normal IgG from the same species. For the MyD88 inhibition assay, RAW264.7 cells were adjusted to 1 × 10^6^ cells/mL and treated with T6167923 (5 μg/mL or 10 μg/mL). After incubation with the cells for 18 h, the cells were stimulated with 1 μg/mL rTs‐HSP70 for 15 min and then collected for Western blot analysis. Since T6167923 was dissolved in DMSO, the control group received an equivalent amount of DMSO to exclude interference.

### 2.13. Animal Immunization and Challenge Schedules

**Figure 1:**
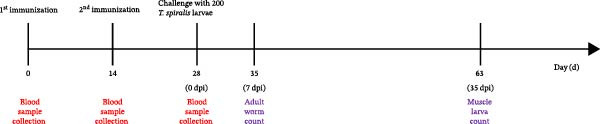
Schematic representation of the vaccination and infection timeline in the study.

PLGA nanoparticles were prepared according to the method described by Yu et al. [26]. Six to 7‐week‐old female ICR mice, weighing ~25 g each, were randomly divided into 3 groups, with 10 mice in each group. On days 0 and 14, each mouse in the experimental group was subcutaneously immunized with PLGA nanoparticles loaded with 100 μg of rTs‐HSP70, while the adjuvant control group was subcutaneously injected with PLGA nanoparticles encapsulating a PBS solution. On day 28, each group of mice was challenged orally with 200 *T. spiralis* larvae. The number of adult worms was counted at 7 days postinfection (dpi), and the number of *T. spiralis* muscle larvae was counted at 35 dpi. The serum collected on days 0, 14, and 28 of the experiment was used to detect specific antibodies and changes in cytokines. A schematic timeline and details of the vaccination/challenge protocol are indicated in Figure 1.

### 2.14. Data Analysis

The gray values of phosphorylated protein, total protein, and the internal reference (GAPDH or tubulin) were quantified using ImageJ. The relative phosphorylation level for each sample was calculated as (phosphorylated protein/internal reference)/(total protein/internal reference). This value was then normalized to the mean of the control group to yield the final fold change for plotting.

Each experiment was performed in three independent replicates, and the data were analyzed using IBM SPSS Statistics 26 software. Differences between two groups were evaluated by an independent sample *t*‐test. Comparisons among multiple groups were performed by one‐way analysis of variance (ANOVA) followed by Duncan’s multiple range test for post‐hoc analysis. Data are presented as the mean ± standard deviation (SD). All graphs were generated using GraphPad Prism 8.

## 3. Results

### 3.1. Co‐IP Confirms the Binding of *T. spiralis* HSP70 to Mouse TLR2

**Figure 2:**
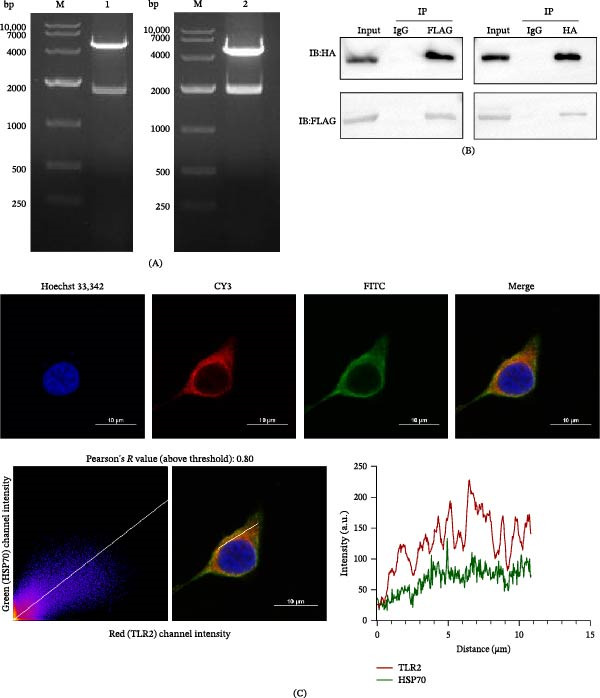
Binding of Ts‐HSP70 to TLR2 (A) Double restriction enzyme digestion analysis of pCAGGS‐TLR2‐HA and pCAGGS‐HSP70‐FLAG plasmids. Lane M: DNA Marker DL10000. Lane 1: Digestion of the pCAGGS‐TLR2‐HA plasmid. Lane 2: Digestion of the pCAGGS‐HSP70‐FLAG plasmid. (B) Verification of the binding between Ts‐HSP70 and mouse TLR2 through Co‐IP assay. (C) Following cotransfection with pCAGGS‐HSP70‐FLAG and pCAGGS‐TLR2‐HA, the subcellular localization of Ts‐HSP70 and the TLR2 extracellular domain in HEK293T cells was observed using confocal laser scanning microscopy. Cell nuclei were stained with Hoechst 33342, TLR2 was labeled with Cy3, and Ts‐HSP70 was detected with FITC, appearing blue, red, and green under their respective excitation wavelengths. Colocalization was analyzed based on fluorescence intensity, with the red curve representing the fluorescence distribution of the TLR2 and the green curve representing the fluorescence distribution of Ts‐HSP70. A Pearson’s correlation coefficient of 0.80, obtained from ImageJ analysis, demonstrated a high degree of spatial colocalization between Ts‐HSP70 (green fluorescence) and TLR2 (red fluorescence).

Double‐enzyme digestion analysis showed a target band of the expected size (Figure 2A), and gene sequencing revealed complete identity between the cloned genes and the target sequences. These results confirmed the successful construction of the pCAGGS‐TLR2‐HA and pCAGGS‐HSP70‐FLAG plasmids.

Following cotransfection, cells were lysed and subjected to Co‐IP using epitope tag‐specific antibodies. As demonstrated in Figure 2B, in the input lanes, recombinant TLR2‐HA protein was detected by anti‐HA antibody, while HSP70‐FLAG was identified by anti‐FLAG antibody. Critically, in anti‐FLAG IP lanes, TLR2‐HA was coprecipitated with HSP70‐FLAG. Similarly, in anti‐HA IP lanes, HSP70‐FLAG was coprecipitated with TLR2‐HA. Additionally, no bands were observed in the IgG control IP lanes, ruling out nonspecific binding. These results conclusively demonstrate that Ts‐HSP70 binds to the mouse TLR2.

### 3.2. Colocalization Analysis

To investigate the subcellular localization, after cotransfection, recombinant proteins were stained with fluorophore‐conjugated tag‐specific antibodies. Quantitative analysis using ImageJ software demonstrated a Pearson’s correlation coefficient of 0.8, indicating a strong spatial association. Fluorescence intensity profiles along a cross‐section showed overlapping distribution patterns of both proteins (Figure 2C).

### 3.3. Recombinant Ts‐Hsp70 Protein Preparation

**Figure 3:**
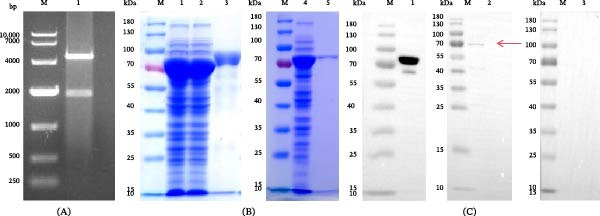
Expression and Western blot identification of rTs‐HSP70. A: Double restriction enzyme digestion analysis of pCold‐HSP70. Lane M: DNA Marker DL10000. Lane 1: identification of pCold‐I double digestion. B: Expression and purification of rTs‐HSP70. Lane M: Protein molecular weight marker. Lane 1: bacterial lysate after induction of expression. Lane 2: soluble proteins in the supernatant of the bacterial lysate. Lane 3: proteins expressed in inclusion bodies from the bacterial lysate. Lane 4: soluble proteins in the supernatant of the bacterial lysate before purification. Lane 5: purified rTs‐HSP70. C: The correct expression of rTs‐HSP70 was verified by Western blot. Lane 1: anti‐His antibody. Lane 2: serum from rats infected with *T. spiralis*. Lane 3: serum from naive rats.

The recombinant plasmid was double‐digested with appropriate restriction enzymes. Double digestion yielded fragments of the expected sizes (Figure 3A, Lane 1). The recombinant protein was expressed in *E. coli* and purified by Ni‐NTA affinity chromatography. SDS‐PAGE revealed that rTs‐HSP70 was successfully expressed in the soluble supernatant fraction (Figure 3B, Lane 2) Following purification, a single band at ~70 kDa was observed (Figure 3B, Lane 5), demonstrating a high‐purity preparation. The protein was further confirmed by Western blot using an anti‐His monoclonal antibody and serum from *T. spiralis*‐infected rats as primary antibodies. The results showed that rTs‐HSP70 was recognized by both the anti‐His antibody and the infected serum, confirming the successful expression and immunoreactivity of rTs‐HSP70 (Figure 3C, Lanes 1 and 2). In contrast, no band was detected when using noninfected rat serum (Figure 3C, lane 3).

### 3.4. Effect of rTs‐HSP70 on the Proliferation, Phagocytic Function, ROS Production, and NO Release in Mouse Macrophages

**Figure 4:**
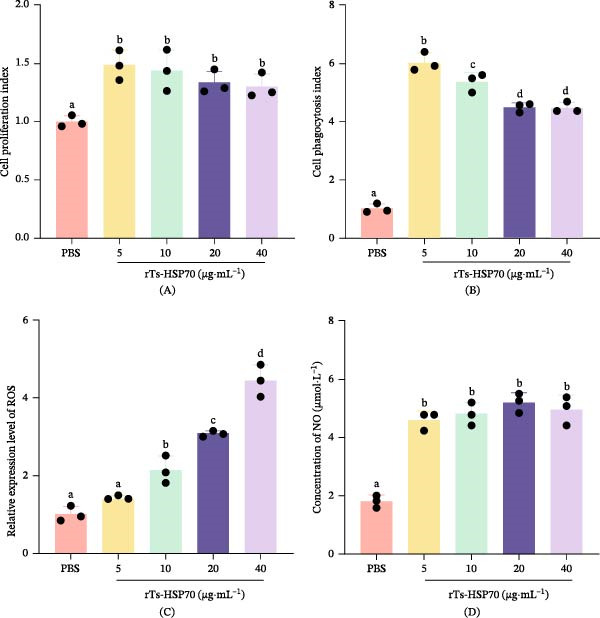
Recombinant Ts‐HSP70 promotes mouse macrophage proliferation, phagocytosis, ROS production, and NO release. After stimulating mouse macrophages with different concentrations of rTs‐HSP70, the changes in cell functions were detected. A. Cell proliferation status. The cell proliferation capacity was evaluated by the proliferation index (absorbance of experimental group/absorbance of PBS control group). B. Cell phagocytic capacity. The phagocytic index (absorbance of experimental group/absorbance of PBS control group) was used to reflect the cell phagocytic function. C. Relative changes in ROS production. D. Amount of NO released by cells. Statistical differences among groups were determined by one‐way ANOVA followed by Duncan’s multiple range test. Different letters above the bars denote statistically significant differences (*p* < 0.05), while the same letters indicate no significant difference.

Exposure of mouse macrophages to rTs‐HSP70 at concentrations of 5, 10, 20, and 40 μg/mL significantly enhanced cellular proliferation and phagocytic activity as compared to the control group (*p*  < 0.05; Figure 4A,B). The most potent proliferative and phagocytic effect was observed at 5 μg/mL (*p*  < 0.05). The ROS production was significantly increased at 10, 20, and 40 μg/mL of rTs‐HSP70 in a concentration‐dependent manner as compared to the control (*p*  < 0.05; Figure 4C). The maximum effect was observed at the highest concentration of 40 μg/mL (*p*  < 0.05). Furthermore, rTs‐HSP70 significantly increased the NO release from mouse macrophages (Figure 4D). All the concentrations induced a significant increase in NO production compared to the control group (*p* < 0.05).

### 3.5. Effects of rTs‐HSP70 on Cytokine mRNA Expression in Mouse Macrophages

**Figure 5:**
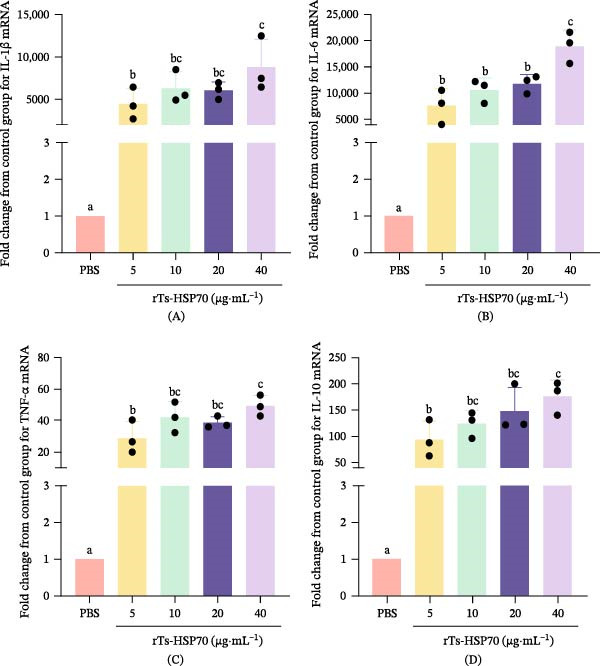
Recombinant Ts‐HSP70 promotes the mRNA expression of inflammatory cytokines. Mouse macrophages were stimulated with 5, 10, 20, or 40 μg/mL rTs‐HSP70 for 6 h. Following stimulation, total cellular RNA was extracted and analyzed by RT‐qPCR. As shown, rTs‐HSP70 upregulated the transcription of IL‐1β (A), IL‐6 (B), TNF‐α (C), and IL‐10 (D) in mouse macrophages. Statistical differences among groups were determined by one‐way ANOVA followed by Duncan’s multiple range test. Different letters above the bars denote statistically significant differences (*p* < 0.05), while the same letters indicate no significant difference.

The results demonstrated that rTs‐HSP70 significantly upregulated the mRNA expression of inflammatory cytokines, including IL‐1β (Figure 5A), IL‐6 (Figure 5B), TNF‐α (Figure 5C), and IL‐10 (Figure 5D), as compared to the control. The most pronounced effect was observed at the concentration of 40 μg/mL (*p*  < 0.05).

### 3.6. Upregulation of TLR2 and MyD88 Expression by rTs‐HSP70 in Mouse Macrophages

**Figure 6:**
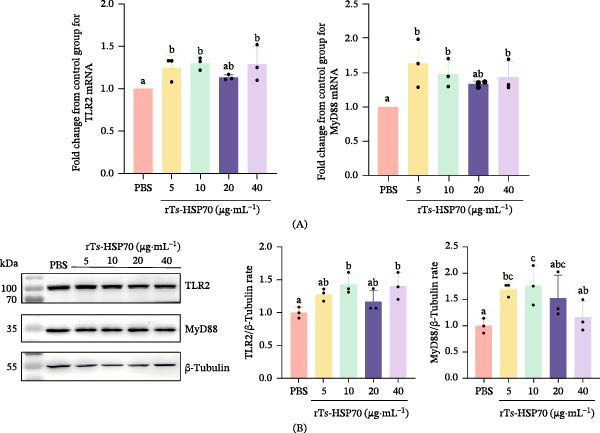
Recombinant Ts‐HSP70 promotes the expression of TLR2 and MyD88. Mouse macrophages were stimulated with 5, 10, 20, or 40 μg/mL rTs‐HSP70 for 12 h. Following stimulation, total cellular RNA and protein were extracted and analyzed. A. The effect of rTs‐HSP70 on the transcription level of TLR2 and MyD88 in mouse macrophage cells. B. Western blot analysis of the TLR2 and MyD88 expression in mouse macrophage cells after rTs‐HSP70 stimulation. Statistical differences among groups were determined by one‐way ANOVA followed by Duncan’s multiple range test. Different letters above the bars denote statistically significant differences (*p* < 0.05), while the same letters indicate no significant difference.

Stimulation of mouse macrophages with rTs‐HSP70 significantly upregulated both the transcriptional and translational expression levels of TLR2 and MyD88, as quantified by qPCR and Western blot analyses, respectively. The mRNA levels of TLR2 and MyD88 were significantly upregulated at 5, 10, and 40 μg/mL of rTs‐HSP70 (*p* < 0.05; Figure 6A). The increase in their protein levels was most pronounced at the concentration of 10 μg/mL (*p* < 0.05; Figure 6B).

### 3.7. Activation of the NF‐κB and MAPK Pathway in Mouse Macrophages

**Figure 7:**
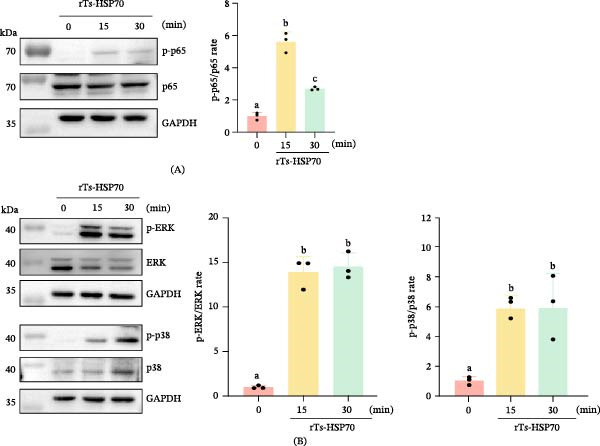
Recombinant Ts‐HSP70 activates the NF‐κB and MAPK signaling pathways. Mouse macrophages were stimulated with 5 μg/mL rTs‐HSP70 for 15 or 30 min. Phosphorylation of p65, ERK, and p38 was analyzed by Western blot. A. NF‐κB pathway activation. B. MAPK pathway activation. Statistical differences among groups were determined by one‐way ANOVA followed by Duncan’s multiple range test. Different letters above the bars denote statistically significant differences (*p* < 0.05), while the same letters indicate no significant difference.

NF‐κB and MAPK are important signaling pathways downstream of TLR2. To evaluate their activation, we examined the phosphorylation levels of NF‐κB p65, ERK, and p38. As shown in Figure 7, stimulation with rTs‐HSP70 significantly increased the phosphorylation levels of NF‐κB p65, ERK, and p38 in mouse macrophages compared to the negative control group (*p* < 0.05). These results suggest that rTs‐HSP70 is capable of activating the NF‐κB and MAPK signaling pathways in macrophages.

### 3.8. Recombinant Ts‐HSP70 Affects Mouse Macrophage Immune Function via the TLR2/MyD88/ERK Signaling Pathway

**Figure 8:**
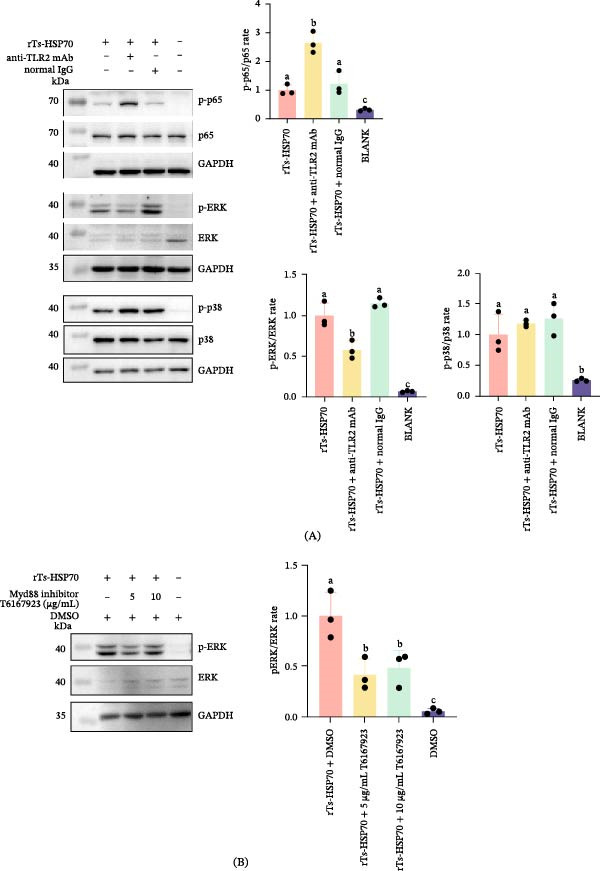
Recombinant Ts‐HSP70 affects mouse macrophage immune function via the TLR2/MyD88/ERK signaling pathway. Mouse macrophages were pretreated with a TLR2‐specific antibody (or control IgG) for 1 h, then stimulated with rTs‐HSP70 (1 μg/mL, 15 min) and harvested for Western blot. In the MyD88 inhibition assay, RAW264.7 cells were pretreated with the MyD88 inhibitor T6167923 (5, 10 μg/mL; DMSO control) for 18 h, then stimulated with rTs‐HSP70 (1 μg/mL, 15 min), and harvested for Western blot analysis. A. TLR2 blocking assay. B. MyD88 inhibition assay. Statistical differences among groups were determined by one‐way ANOVA followed by Duncan’s multiple range test. Different letters above bars indicate significant differences (*p* < 0.05); the same letter indicates no significant difference.

The results showed that compared with the normal IgG treatment group, blocking TLR2 with a specific antibody significantly downregulated ERK phosphorylation activated by rTs‐HSP70 (*p* < 0.05). This indicates that rTs‐HSP70 may activate the ERK pathway through the TLR2 pathway. For the p38 pathway, there was no significant difference between the TLR2‐specific antibody blockade and the normal IgG treatment, suggesting that rTs‐HSP70 may activate p38 through other related pathways rather than TLR2. Interestingly, after blocking TLR2 with the specific antibody, the activation of NF‐κB p65 by rTs‐HSP70 was enhanced, and we detected a higher degree of p65 phosphorylation compared with the normal IgG treatment (Figure 8A).

Furthermore, for the ERK pathway, treatment of cells with the MyD88 inhibitor T6167923 significantly downregulated the phosphorylation level of ERK, whereas the solvent DMSO alone did not cause such downregulation (Figure 8B).

These findings indicate that rTs‐HSP70 may significantly affect host immunity through the TLR2/MyD88/ERK pathway.

### 3.9. Preparation of Nanoparticles and Immune Protection Assay

**Figure 9:**
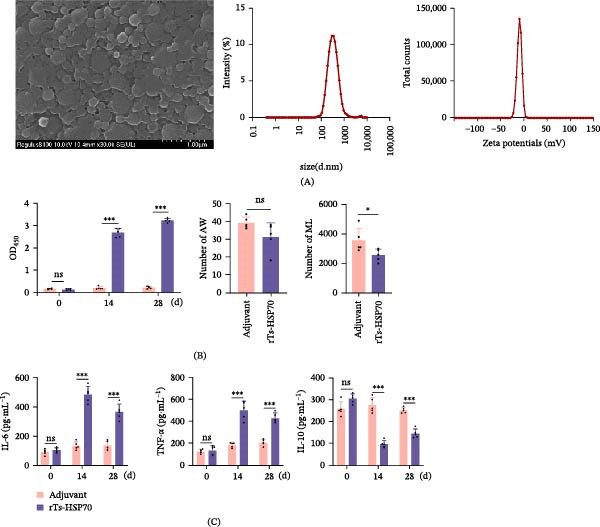
Preparation of rTs‐HSP70 PLGA nanoparticles and immune protection assay. Mice were subcutaneously immunized with 100 μg HSP70 PLGA nanoparticles on days 0 and 14. Two weeks after final immunization, animals were orally infected with 200 *T. spiralis* larvae. Adult worms were collected and enumerated at 7 dpi, while muscle larvae burdens were assessed at 35 dpi. Serum samples were collected for the determination of specific IgG antibodies and the cytokines IL‐6, TNF‐α, and IL‐10. A. SEM images, DLS, and zeta potential of rTs‐HSP70 PLGA nanoparticles. B. Specific antibody levels and worm burden reduction efficacy. C. Changes in inflammatory cytokines in mice following immunization with rTs‐HSP70 PLGA nanoparticles. A *t*‐test was used to analyze the significance of differences between groups. Differences were significantly indicated by  ^∗^ (*p*  < 0.05),  ^∗∗∗^ (*p*  < 0.001); differences were not significant, indicated by “ns” (*p* > 0.05).

The PLGA rTs‐HSP70 nanoparticles were examined by scanning electron microscopy, and the encapsulation efficiency was 76.6%. The dynamic light scattering (DLS) mean size was 292.7 ± 3.2 nm, the polydispersity index (PDI) was 0.23 ± 0.02, and the zeta potential was –9.49 ± 0.24 mV (Figure 9A).

Immunization with rTs‐HSP70 nanoparticles induced high levels of specific antibodies and provided a certain degree of immune protection to mice, resulting in a significant 28.0% reduction in the burden of *T. spiralis* muscle larvae (*p* < 0.05) and a 20.4% reduction in adult worm burden, although the latter was not statistically significant (*p* > 0.05; Figure 9B). It also significantly enhanced the secretion of IL‐6 and TNF‐α while suppressing the production of IL‐10 (*p* < 0.05; Figure 9C).

## 4. Discussion

In the present study, we demonstrate that Ts‐HSP70 interacts with TLR2 and modulates macrophage immune functions. Stimulation with rTs‐HSP70 enhanced macrophage proliferation, phagocytosis, and ROS/NO production and was associated with activation of NF‐κB and MAPK signaling pathways. Functional inhibition assays further suggested that ERK activation is at least partially dependent on the TLR2/MyD88 signaling axis. In addition, immunization with rTs‐HSP70 encapsulated in PLGA nanoparticles elicited antigen‐specific immune responses and conferred partial protection against *T. spiralis* infection in mice.

It is well established that HSP70 is not only present inside cells but can also be released into the extracellular space to exert functions. The functional effects of HSP70 are diverse and context‐dependent and are particularly closely related to its localization. HSP70 has completely different or even opposite roles inside and outside cells. Intracellular HSP70 (iHSP70) generally exerts anti‐inflammatory functions, suppressing inflammatory responses and protecting cells from excessive immune damage [27, 28]. In contrast, extracellular HSP70 (eHSP70), whether actively secreted or passively released, often acts as a danger signal or PAMP‐like molecule, inducing the expression of proinflammatory cytokines and initiating or amplifying immune responses [20]. For example, in sepsis, eHSP70 promotes the polarization of macrophages toward the M1 phenotype and the release of pro‐inflammatory cytokines through the TLR2/MyD88/NF‐κB pathway [29]. Similarly, the HSP70‐like protein DnaK secreted by *Pseudomonas aeruginosa* induces IL‐1β expression via TLR4 [30]. In contrast, iHSP70 in tumor cells can upregulate the MerTK receptor through TLR2, inducing macrophage polarization toward the M2 phenotype and promoting tumor cell escape and proliferation [31]. Furthermore, the traditional Chinese medicine Yangxue Jiedu Soup alleviates psoriasis symptoms by inhibiting the secretion of HSP70, thereby suppressing NF‐κB pathway activation and reducing the release of inflammatory factors [32].

In fact, we had previously screened Ts‐HSP70 from *T. spiralis* excretory‐secretory proteins, and in this study, we exogenously added rTs‐HSP70. Therefore, the series of proinflammatory effects we observed, such as enhanced macrophage proliferation, increased phagocytic capacity, elevated NO/ROS production, activated NF‐κB and MAPK pathways, and upregulation of proinflammatory cytokine mRNAs, are consistent with the classical functions of eHSP70. We found that blocking TLR2 with a specific antibody significantly reduced HSP70‐induced activation of the ERK pathway, and treatment with a MyD88 inhibitor also markedly downregulated ERK phosphorylation. Based on these results, we speculate that Ts‐HSP70 may influence macrophage functions through the TLR2/MyD88/ERK signaling pathway. However, unexpectedly, blocking TLR2 led to increased phosphorylation of NF‐κB p65, which is contrary to the previous findings. Although this result cannot be fully explained at present, it suggests that the activation of NF‐κB by rTs‐HSP70 may not depend on a single receptor but rather on a redundant, multireceptor network. Future studies should consider the involvement of other receptors, particularly TLR4. It has been repeatedly reported that HSP70 can activate downstream pathways via the TLR4. Therefore, further validation is necessary using a TLR2/TLR4 double‐gene knockout model.

Research on vaccines against *T. spiralis* has been ongoing for several decades; however, to date, no commercialized vaccine is available. The complex and variable antigens of *T. spiralis* represent a major obstacle to vaccine development. HSP70 is a highly conserved protein across all organisms and often functions as a molecular chaperone, having gained widespread attention in vaccine development [33]. Mohamed et al. [34] demonstrated that vaccination with a DNA vaccine encoding the *Toxoplasma gondii* HSP70 gene significantly reduced the parasite burden in various organs. Carrión et al. [35] constructed a *Leishmania* mutant strain with a disrupted HSP70‐II gene, which served as a safe live attenuated vaccine and conferred protective immunity in mice. Furthermore, Ts‐HSP70 promotes DC maturation via the TLR2 and TLR4 signaling pathways, thereby exerting an immunoprotective effect [23]. Our in vivo experiments also confirmed that Ts‐HSP70 induces an immune response in the host, characterized by high levels of specific antibodies, upregulation of the proinflammatory cytokines IL‐6 and TNF‐α, and a certain degree of protective immunity, ultimately leading to a reduction in the larval burden in mice. Notably, in contrast to the in vitro findings, IL‐10 production was decreased in vivo. In the in vitro setting, macrophages were directly stimulated by rTs‐HSP70, resulting in a relatively simple and direct signaling pathway. In contrast, during in vivo immunization, multiple immune cell types (such as DCs, T cells, and B cells) and a complex cytokine network collectively shape the immune response. IL‐10 is a regulatory cytokine that mitigates inflammation‐induced tissue damage by suppressing excessive immune responses. IL‐10 plays a complex role in parasitic infections. Studies on intestinal nematodes have shown that IL‐10 can indirectly promote parasite expulsion [36, 37]. Although *T. spiralis* is not a strictly intestinal parasite; its life cycle includes an intestinal phase, which may partially account for the suboptimal efficacy of the vaccine.

Wang et al. [21] reported that the HSP70 subunit vaccine achieved a worm reduction rate of 37% against muscle larvae in mice. In the present study, our prepared HSP70 PLGA nanoparticles exhibited a 20.4% reduction in adult worm burden (not statistically significant) and a 28.0% reduction in muscle larval burden (*p* < 0.05). Although we optimized the expression of rTs‐HSP70 protein in a soluble form, the protective efficacy of the recombinant Ts‐HSP70 PLGA nanovaccine was limited and inferior to that of the subunit vaccine adjuvanted with Freund’s adjuvant. Several factors may account for this outcome. First, the preparation process of PLGA nanoparticles requires further optimization. DLS analysis showed that the nanoparticles had an average size of 292.7 ± 3.2 nm, a PDI of 0.23 ± 0.02, and a zeta potential of −9.49 ± 0.24 mV. The relatively low zeta potential indicates suboptimal particle stability, and scanning electron microscopy revealed an uneven particle morphology. Second, the immunization regimen and route of administration were not systematically optimized in this study. Furthermore, PLGA nanoparticles and Freund’s adjuvant may differ in the types of immune responses they induce. Collectively, these factors may affect the protective efficacy of the vaccine. Despite these limitations in morphology and stability, the prepared PLGA nanoparticles still essentially met the experimental requirements for in vivo *s*tudies and induced high levels of specific antibodies.

Neither previous studies nor the present investigation has shown particularly ideal protective efficacy of HSP70 alone as a candidate vaccine antigen. In addition to being used directly as a vaccine or diagnostic antigen, HSP70 can also serve as a fusion protein adjuvant or immunomodulator to enhance the host’s immune response against an antigen. Relevant studies have already been reported. Jiang et al. [38] demonstrated that mice immunized with a target antigen fused with HSP70 produced significantly higher specific antibody titers, IFN‐γ levels, and cytotoxic T‐lymphocyte (CTL) activity compared to groups receiving the antigen alone or a simple mixture of the antigen and HSP70. Similarly, fusion of a target gene with the HSP70 gene in a DNA vaccine enhanced the protective efficacy through a CD8^+^ T cell‐dependent pathway [39]. The C‐terminal domain of *Mycobacterium tuberculosis* HSP70 (amino acids 359–610) has been shown to enhance immune responses [40], providing a structural basis for the subsequent design of fusion proteins combining a target gene with Ts‐HSP70. Furthermore, studies have shown that Ts‐HSP70 promotes DC maturation and induces T‐cell proliferation and differentiation. The present study confirmed that it activates the NF‐κB and MAPK signaling pathways and promotes the inflammatory response of macrophages. These characteristics are exactly what an ideal adjuvant should possess. Therefore, in future vaccine strategies against *T. spiralis*, we recommend fusing Ts‐HSP70 with antigens of greater protective potential, enabling it to serve dual functions as a candidate antigen and an adjuvant.

## 5. Conclusion

In conclusion, Ts‐HSP70 modulates macrophage immune functions and activates the NF‐κB and MAPK signaling pathways. Our findings suggest that Ts‐HSP70 may exert immunomodulatory effects through TLR2‐associated signaling and contribute to host defense against *T. spiralis* infection. These results provide a foundation for further exploration of Ts‐HSP70 as a potential vaccine antigen and an immunomodulatory component.

## Author Contributions

Ruofeng Yan conceived and designed the study.Jiajun Feng performed the experiments, analyzed the data, and drafted the manuscript. Yuan Li, Muhammad Azhar Memon, and Yuheng Zhang assisted with the experiments and data analysis. Mingmin Lu, Xiaokai Song, and Lixin Xu supervised the research and provided critical guidance.

## Funding

This work was supported by the Social Development Project of Jiangsu Province (Grant BE2022848) and a project funded by the Priority Academic Program Development of Jiangsu Higher Education Institutions (PAPD).

## Disclosure

All authors reviewed the manuscript and approved the final version.

## Conflicts of Interest

The authors declare no conflicts of interest.

## Data Availability

The data that support the findings of this study are available from the corresponding author upon reasonable request.
